# Supplementary material for: Optimal minimal residual disease threshold in pediatric acute myeloid leukemia: A retrospective cohort study based on the TARGET database
Source: PLoS Med. 2026 May 8;23(5):e1005088. doi: 10.1371/journal.pmed.1005088 (PMC13155632; doi:10.1371/journal.pmed.1005088)
Supplement: S1 Code — (ZIP) [file pmed.1005088.s002.zip › S2 code/PROJ8_1_tbl1/PROJ8_1_tbl1_full_nom.pdf]

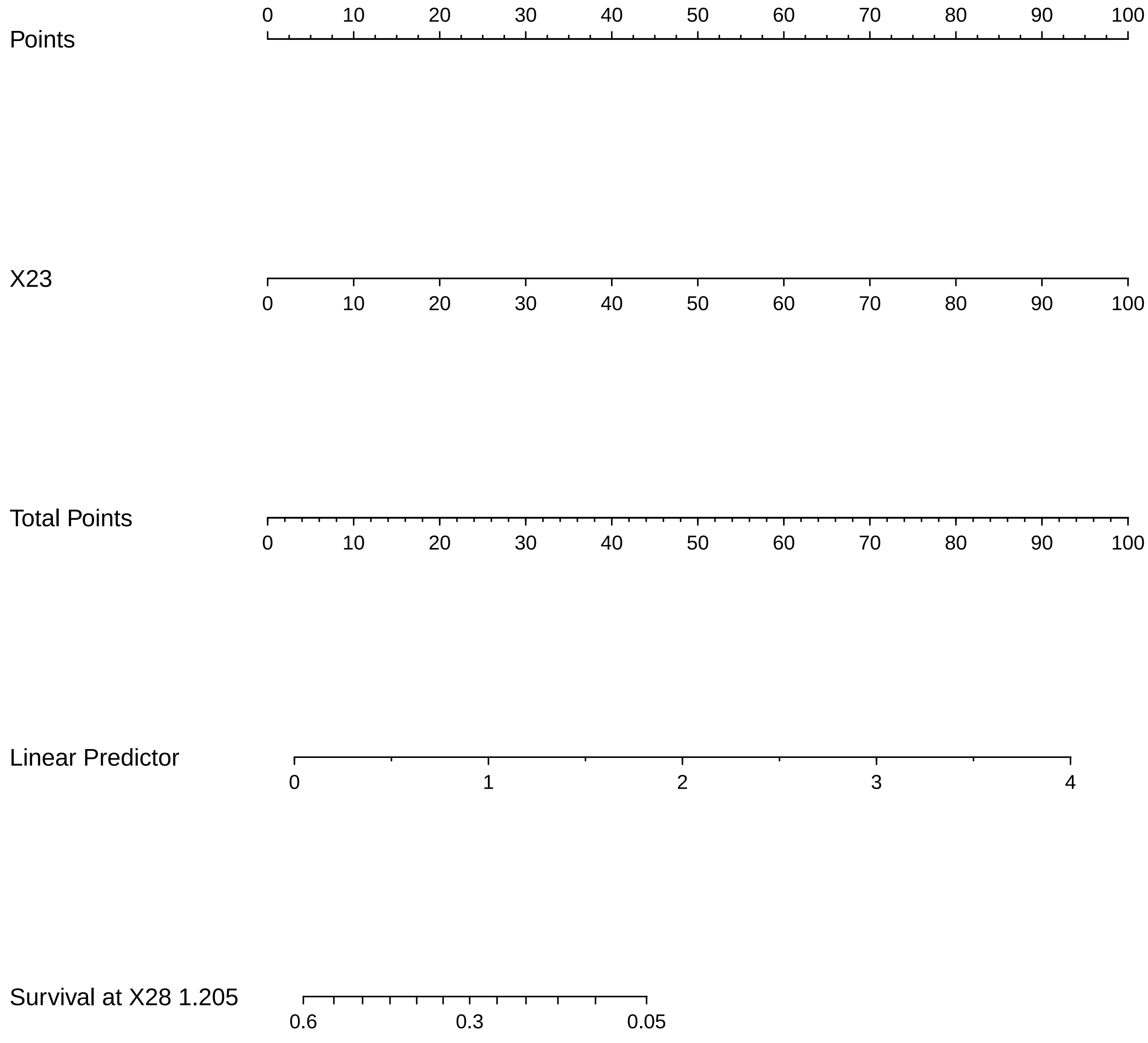
Points
0 10 20 30 40 50 60 70 80 90 100
X23
0 10 20 30 40 50 60 70 80 90 100
Total Points
0 10 20 30 40 50 60 70 80 90 100
Linear Predictor
0 1 2 3 4
Survival at X28 1.205
0.6 0.3 0.05
